# Low prevalence of lipodystrophy in HIV-infected Senegalese children on long-term antiretroviral treatment: the ANRS 12279 MAGGSEN Pediatric Cohort Study

**DOI:** 10.1186/s12879-018-3282-7

**Published:** 2018-08-06

**Authors:** Cecile Cames, Lea Pascal, Aissatou Ba, Hélène Mbodj, Baly Ouattara, Ndeye-Fatou Diallo, Philippe Msellati, Ngagne Mbaye, Haby Sy Signate, Stephane Blanche, Aminata Diack, for the MAGGSEN Cohort Study Group, Haby Sy Signate, Aminata Diack, Helene Mbodj, Ndeye Fatou Diallo, Aissata Ba, Aicha Dia, Ndeye Ngone Have, Astou Dieye, Oumy Fall, Rosine Sonko, Ngagne Mbaye, Baly Ouattara, Abdou K. Niang, Alhadji B. Diom, Adama Ndour, Bigue Badiane, Cecile Cames, Lea Pascal, Philippe Msellati, Caroline Desclaux-Sall, Suzanne Izard, Amady Ndiaye, Ousseynou Ndiaye, Binta Seck

**Affiliations:** 1 Institut de Recherche pour le Développement (IRD), UMI233 IRD, INSERM U1175, Université de Montpellier, 911, avenue Agropolis, BP 64501, 34394 Montpellier cedex 5, France; 2 Centre Hospitalier National d’Enfants Albert Royer, Dakar, Sénégal; 3 Synergie Pour l’Enfance, Centre Hospitalier Roi Baudouin, Guediawaye, Sénégal; 4 Hôpital Necker Enfants Malades, Paris, France

**Keywords:** Lipodystrophy, Lipoatrophy, Children, Adolescents, HIV-infection, ART, Stavudine, Zidovudine, Protease inhibitor

## Abstract

**Background:**

The long-term benefits of antiretroviral treatment (ART) are associated with metabolic complications, especially lipodystrophy, which has been well described among HIV-infected adults and children on ART in developed settings. Specifically, stavudine, and to a lesser extent zidovudine and protease inhibitors (PI), have been consistently implicated in the development of lipodystrophy. In 2006, following advice from the WHO, Senegal began phasing out stavudine from first-line ART. The objectives of this cross-sectional analysis are to assess and identify risk factors affecting the prevalence of lipodystrophy in Senegalese children and adolescents on long-term ART participating in a cohort study.

**Methods:**

Lipodystrophy was clinically assessed in two- to 18-year-old children on ART for at least six months and with no concurrent severe acute malnutrition. Risk factors for lipodystrophy were identified using stepwise multivariable logistic regression. Explanatory variables included clinical and personal data, immunovirologic status, and therapeutic history.

**Results:**

Overall, 254 children were assessed for lipodystrophy. The median age was 10.9 years (IQR: 8.1–14.2) and the median duration on ART was 54 months (32–84). Only 18% had been previously treated with stavudine, with a median treatment duration of 8 months (5–25). Ongoing treatment included 76% of children receiving zidovudine (median duration of 48 months (26–74)) and 27% receiving PI (lopinavir/ritonavir; median duration of 49 months (23–59)). Mild signs of lipodystrophy were observed in 33 children (13%): 28 with lipoatrophy, 4 with lipohypertrophy and one with combined type. Boys were more likely to present with lipoatrophy than girls (aOR: 4.3, 95% CI: 1.6–11.7). Children previously treated with stavudine for ≥1 year had a greater risk for lipoatrophy than those never exposed (3.8, 1.0–14.0), although the association was weak. There was no association between lipodystrophy and age or current or cumulative treatment with lopinavir/ritonavir or zidovudine.

**Conclusions:**

We report low prevalence of mild lipodystrophy in children and adolescents on long-term ART receiving a stavudine-sparing regimen. These findings are reassuring for clinicians in low-income settings where zidovudine is massively prescribed and lopinavir/ritonavir is the only widely available PI.

**Trial registration:**

ClinicalTrials.gov identifier: NCT01771562 (registration date: 01/18/2013).

## Background

The long-term benefits of antiretroviral treatment (ART) are associated with metabolic complications, especially lipodystrophy, which has been well described among HIV-infected adults and children on ART. Lipodystrophy diagnosis involves changes in regional fat distribution, including peripheral fat loss (lipoatrophy), central fat accumulation (lipohypertrophy), or a combination of both. Because lipodystrophy, and in particular the potentially disfiguring effects of lipoatrophy, may negatively affect treatment adherence and effectiveness [1], such effects are of major concern in children and adolescents who may require life-long ART.

In studies published over the past decade, the prevalence of lipodystrophy in children receiving ART has ranged widely, from 10 to 84% [2–9], depending on the detection method. Stavudine, and to a lesser extent zidovudine, both of which are thymidine-analogue nucleoside reverse transcriptase inhibitors (NRTI), have consistently been associated with lipoatrophy, affecting between 8 to 28% of children, after 2–5 years of treatment [2, 3, 7, 10]. Protease inhibitors (PI) have been previously implicated in the development of both fat loss and accumulation [11, 12], thus complicating the study of risk factors, as these drugs may be administered together. Lastly, lipodystrophy development is likely multifactorial, including other risk factors, such as puberty [5, 6, 13], ethnicity [2, 3], HIV disease progression [3, 8] and host factors [14].

Compared to high to middle-income settings, much less is known about lipodystrophy in African children and especially adolescents. Two recent cross-sectional studies in Uganda and Tanzania reported mild lipoatrophy in 27% [13] and 30% [15] of children aged one to 18 years, after substantial stavudine exposure. Conversely, moderate to severe lipoatrophy was reported in 36% of prepubescent South African children following a median treatment duration of 41 months with stavudine [16]. Data on fat distribution abnormalities among HIV-infected children and adolescents in other regions of Africa are unavailable.

A 2006 World Health Organization (WHO) report highlighted stavudine-associated toxicities and advised that stavudine be substituted with abacavir in patients with lipoatrophy [17]. In response, from 2007 to 2008 the Senegalese National Program phased out stavudine from first-line pediatric treatment. Due to the prohibitive cost of abacavir, most children were switched to zidovudine. Thereafter, stavudine was used sparingly until 2012, due to lack of alternative options in some patients or as a substitution drug for six months in children with severe anemia. In 2015, it was estimated that 4800 (4000–5600) children aged 0 to 14 years were living with HIV in Senegal, of whom 26% (19–31%) were receiving ART [18].

The ANRS 12279 Maggsen Pediatric Cohort Study aims to investigate growth and metabolic disorders among HIV-infected Senegalese children and adolescents on long-term ART and under active follow-up at the country’s two earliest and most important pediatric HIV clinics, Albert Royer and Roi Baudouin Hospitals, which account for 30% of all children on ART at the country level. Our present objectives are to characterize and assess the prevalence of lipodystrophy and to identify risk factors in cross-sectional analyses conducted at mid-term of the cohort study.

## Methods

### Study population

Full details of the rationale for the Maggsen Pediatric Cohort Study and methods are provided elsewhere [19]. Briefly, the study includes HIV-1-infected children aged two to < 16 years under active follow-up in two Senegalese HIV clinics from April 2013. Children were seen every three months for a complete clinical assessment, and every six months for laboratory monitoring/fasting blood analyses. The present cross-sectional analysis includes study patients under active follow-up from May to December 2015, who had at least six months on ART. As it might be difficult to distinguish between lipoatrophy and wasting syndrome, children experiencing severe acute malnutrition were excluded from the analysis. Severe acute malnutrition is defined for both weight-for-height z scores (WHZ) in children < 5 years or body mass index-for-age z score (BMIZ) in children ≥5 years as being <− 3 [20, 21].

### Outcomes

In May 2015, four pediatricians at the clinics (three from Albert Royer Hospital and one from Roi Baudouin Hospital) received practical and collective training for lipodystrophy diagnosis followed by clinical assessment of 102 children in the cohort. These sessions were conducted and supervised by a senior researcher and pediatrician (SB) experienced in pediatric lipodystrophy, who was both blind to children’s ART regimen and independent from the cohort study. Diagnosis of the remaining children was then assigned to the permanent pediatrician at each site and performed when the children came to their scheduled visit. Lipoatrophy was defined as fat loss in one or more of the following sites: face, arm, leg, or buttocks. Lipohypertrophy was defined as fat gain in one or more of the following sites: abdomen, neck, or breast. Children with at least one sign of lipoatrophy and one sign of lipohypertrophy were defined as combined type [7]. Body fat redistribution was rated as none – no fat changes; mild – possible minor changes, noticeable only on close inspection; moderate – moderate changes, readily noticeable to an experienced clinician or a close relative who knows the child well; severe – major changes, readily noticeable to a casual observer [22]. Clinical assessment used a scoring sheet included in the child’s medical file. At Albert Royer Hospital, in case of doubt between a grade mild and a grade moderate, or a grade moderate and grade severe, the procedure was to require an additional diagnosis from another trained pediatrician and if discordant, to grade at the lower score. At Roi Baudouin Hospital, however, there was only one pediatrician in charge of HIV care and grades were assigned at their discretion.

### Variables and definitions

Data recorded from medical files included WHZ or BMIZ scores, puberty onset (defined as a child reaching stage 2 of the Tanner classification [23]), WHO stage at ART initiation and therapeutic history, CD4 cell counts and viral load (undetectable defined as HIV RNA < 40 copies per milliliter), total cholesterol, LDL cholesterol and triglycerides. Dyslipidemia was defined as total cholesterol and/or isolated HDL and/or triglycerides abnormalities. Clinical and laboratory variables were those collected just prior to the lipodystrophy assessment visit (< 3 and < 6 months, respectively).

### Data analysis

The primary outcomes investigated were fat redistribution (any form) and lipoatrophy. Children characteristics including, age, sex, puberty onset, nutritional status, WHO stage at ART initiation, stavudine, zidovudine and PI treatments and durations, CD4 count, virologic suppression and lipid profiles were compared with the primary outcomes. For each ART drug, recent drug treatment (defined as current treatment or treatment ending within the previous six months), any treatment (defined as drug ever used) and exposure duration were included as separate variables. Associations from univariable analyses were assessed using the Kruskal-Wallis and Wilcoxon rank-sum tests, Chi-square and Fisher’s exact, and univariable logistic regression, as appropriate. Risk factors for the primary outcomes were identified using a stepwise multivariable logistic regression. Explanatory variables with *P* < 0.25 in univariable analysis were included in the multivariable models and exited by the stepwise procedure at *P* ≥ 0.20. Continuous explanatory variables were categorized before entering the models.

Continuous variables are expressed as median with interquartile range (IQR). Odds ratios (OR) and adjusted OR (aOR) are given with their 95% confidence interval (CI) in univariable and multivariable analyses, respectively. Differences were considered statistically significant at *P <* 0.05. All statistical analyses were performed in SAS version 9.3 (SAS Institute, Cary, North Carolina, USA).

### Ethics

Ethics clearance for the cohort study was given by the Ethics and Regulatory Committees in Senegal and the Ethics Committee of the Institute of Research for Development in France. All parents or legal guardians provided written informed consent on behalf of their children. The cohort was registered with ClinicalTrials.gov: NCT01771562 on 01/18/2013.

## Results

### Size and characteristics of the study population

Between May and December 2015, among 335 children included in the cohort, 323 were still in care in the study facilities. Of these, 42 children were on ART for less than six months and 27 presented with severe acute malnutrition. Therefore, the study population for this analysis comprised 254 patients (Table 1). The median age was 10.9 years (8.1–14.2). Overall, 74% of children were on first-line ART, 26% on second-line ART and one child was on third-line ART. There were no differences between boys and girls with regard to therapeutic and immunovirologic data, however, boys were more likely than girls to present moderate wasting (33% vs 14%, *P* = 0.0007) and prepubescent status (72% vs 50%, *P* = 0.0004).

**Table 1:** Characteristics of HIV-infected children on ART in the Maggsen Cohort Study.^a–b^ Dakar, Senegal

Characteristics	2–5 y		5–10 y		10–18 y		Total		*P* value
	(*N* = 18)		(*N* = 83)		(*N* = 153)		(*N* = 254)		
Study site									0.79
Albert Royer Hospital	13	(72)	61	(73)	106	(69)	180	(71)	
Roi Baudouin Hospital	5	(28)	22	(27)	47	(31)	74	(29)	
Girls	6	(33)	37	(45)	68	(44)	111	(44)	0.65
WHO classification^c^									0.03
Stage 1–2	8	(44)	14	(17)	29	(19)	51	(20)	
Stage 3–4	10	(56)	69	(83)	124	(81)	203	(80)	
Moderate wasting^d^	0	–	14	(17)	49	(32)	63	(25)	0.002
WHZ/BMIZ, median (IQR)	−0.7	(−1.6–0.2)	−1.1	(−1.8 – −0.4)	−1.6	(−2.2 – − 0.9)	−1.4	(−2.0 – − 0.7)	< 0.0001
HAZ, median (IQR)	− 1.3	(−2.0 – − 1.0)	−1.1	(− 1.7 – − 0.3)	−1.2	(− 1.9 – − 0.5)	−1.2	(− 1.8 – − 0.5)	0.37
Puberty onset	0	–	0	–	92	(60)	92	(36)	< 0.0001
CD4 < 500 cells/mm^3^	2	(11)	7	(9)	38	(25)	47	(19)	0.01
Viral load < 40 copies/mL	7	(39)	57	(70)	108	(71)	172	(68)	0.02
Time on ART in month, median (IQR)	22	(11–30)	47	(31–61)	64	(34–100)	54	(32–84)	< 0.0001
ARV drug classes									
2NRTI/NNRTI	15	(83)	65	(78)	72	(47)	152	(60)	
2NRTI/PI	3	(17)	15	(18)	28	(18)	46	(18)	
NRTI/NNRTI/nRTI	0	–	1	(1)	30	(20)	31	(12)	
NRTI/nRTI/PI	0	–	2	(3)	22	(14)	24	(10)	
NRTI/nRTI/PI/II	0	–	0	–	1	(1)	1	(0)	

^a^Abbreviations: *ART* antiretroviral treatment, *BMIZ* body mass index for age z-score, *HAZ* height for age z-score, *II* integrase inhibitor, *nRTI* nucleotide reverse-transcriptase inhibitor, *NRTI* nucleoside reverse-transcriptase inhibitor, *NNRTI* non-nucleoside reverse-transcriptase inhibitor, *IQR* interquartile range, *PI/r* protease inhibitor boosted, *RTV* ritonavir, *WHZ* weight for height z-score

^b^Data are N (%) unless otherwise indicated

^c^WHO symptoms classification: highest stage reached by the child before ART initiation

^d^Moderate wasting (moderate acute malnutrition) is defined for both weight-for-height z scores (WHZ) in children < 5 years or body mass index-for-age z score (BMIZ) in children ≥5 years as being ≥ − 3 and < −2 [20, 21]

### Prevalence and characterization of fat redistribution

Overall, 33 cases (13%) of lipodystrophy were clinically reported, all in children older than five years of age. Signs of fat loss were observed in 28 children (11%), signs of fat accumulation in four children (2%), and only one child presented with combined type (Table 2). All signs of fat redistribution were graded “mild” (grade 1). While there is often inherent uncertainty in grading mild lipodystrophy, there were no examples of doubt in a clinical diagnosis between mild versus moderate (grade 2) or severe (grade 3) signs of lipodystrophy reported in the study.

**Table 2:** Metabolic abnormalities in HIV-infected children on ART in the Maggsen Cohort Study.^a–b^ Dakar, Senegal

Characteristics	Boys		Girls		Total		*P* value
	(*N* = 143)		(*N* = 111)		(*N* = 254)		
Lipodystrophy^c^	25	(17)	8	(7)	33	(13)	0.02
Lipoatrophy	24	(17)	4	(4)	28	(11)	–
Lipohypertrophy	1	(1)	3	(3)	4	(2)	–
Combined type	0	–	1	(1)	1	(0)	–
Dyslipidemia	30	(21)	33	(30)	63	(25)	0.11
Abnormal total cholesterol (> 200 mg/dL)	28	(20)	31	(28)	59	(23)	0.11
Abnormal LDL cholesterol (> 130 mg/dL)	18	(13)	25	(23)	43	(17)	0.04
Abnormal triglycerides (> 150 mg/dL)	3	(2)	4	(4)	7	(3)	0.47

^a^Abbreviations: *ART* antiretroviral treatment, *LDL* low-density lipoprotein

^b^Data are N (%)

^c^All cases were graded “mild”

In 28 children with signs of lipoatrophy, 22 presented with only prominent veins in the arms while the remaining six presented with additional signs of fat loss (six with sunken cheeks, two with prominent veins in the legs and two with sunken buttocks). Abdominal fat accumulation was present in the four children with lipohypertrophy. Fat redistribution was not associated with age categories (< 10 and ≥ 10 years) or any lipid abnormality.

### Fat redistribution by treatment with ART drugs

Signs of lipodystrophy were weakly associated with prior treatment with stavudine and longer PI treatment duration (Table 3). Few children (18%) had been previously treated with stavudine and for a limited median treatment duration of 8 months (5–25). Stavudine treatment in these children could only have occurred at any time between September 2003 and November 2012, so that at enrollment in the cohort study, no child was still receiving stavudine. Almost all children had been treated with zidovudine, and 76% were currently receiving this ART drug in their backbone regimen, for a median treatment duration of 48 months (26–74). The median duration of current treatment with PI, essentially lopinavir/ritonavir (lopinavir/r), was 49 months (23–59). Treatment with other PI was marginal: six children had previously received a nelfinavir-based ART for a median duration of 61 months (53–87) and eight were currently treated with an atazanavir/ritonavir-based regimen.

**Table 3:** Univariable regression analysis of potential risk factors for fat abnormalities in the Maggsen cohort study^a^, Dakar, Senegal

Characteristics	Lipoatrophy			Fat abnormality		
	OR	95% CI	*P* value	OR	95% CI	*P* value
Boys	4.3	1.6–11.6	0.004	2.7	1.2–6.3	0.02
Age ≥ 10 years	1.3	0.6–2.9	0.54	1.4	0.6–3.0	0.42
Puberty onset: Yes	1.2	0.5–2.7	0.64	1.3	0.6–2.7	0.52
Moderate wastingb: Yes	1.7	0.7–3.9	0.20	1.4	0.6–3.1	0.43
WHO classification Stage^c^ 3/4 vs 1/2	1.7	0.5–5.0	0.37	2.0	0.7–5.8	0.23
Viral load > 40 copies/mL: Yes	1.1	0.5–2.6	0.76	0.9	0.4–2.0	0.82
CD4 < 500 cells/mm^3^: Yes	0.5	0.1–1.7	0.26	0.6	0.2–1.8	0.34
Stavudine ever used: Yes	2.1	0.9–5.2	0.09	2.1	0.9–5.0	0.08
Stavudine exposure 1 year vs No	1.5	0.5–4.8	0.48	1.7	0.6–4.9	0.32
≥1 year vs No	3.6	1.0–12.6	0.04	3.1	0.9–10.8	0.07
Zidovudine recent: Yes	1.0	0.4–2.4	0.94	1.0	0.4–2.3	0.93
Zidovudine exposure < 3 years vs No	0.5	0.2–1.8	0.30	0.6	0.2–1.7	0.30
≥3 years vs No	1.3	0.5–3.3	0.61	1.2	0.5–3.0	0.63
Lopinavir/r recent: Yes	1.3	0.6–3.1	0.51	1.1	0.5–2.5	0.86
Lopinavir/r exposure < 3 years vs No	0.3	0.0–2.5	0.28	0.3	0.0–2.0	0.20
≥3 years vs No	2.2	0.9–5.4	0.09	1.8	0.7–4.3	0.21

^a^Abbreviations: *CI* confidence interval, *Lopinavir/r* lopinavir/ritonavir, *OR* odds ratio

^b^Moderate wasting (moderate acute malnutrition) is defined for both weight-for-height z scores (WHZ) in children < 5 years or body mass index-for-age z score (BMIZ) in children ≥5 years as being ≥ − 3 and < − 2 [20, 21]

^c^WHO symptoms classification: highest stage reached by the child before ART initiation

### Factors associated with lipoatrophy

To identify risk factors for lipoatrophy, we ran a stepwise logistic regression model with ‘lipoatrophy + combined type’ as the dependent variable. Univariable analyses identified sex, moderate wasting, exposure to stavudine (no vs. < 1 year vs. ≥ 1 year), and recent exposure to lopinavir/r (no vs. < 3 years vs. ≥ 3 years) for inclusion in the model (Table 4). Forcing age (< 10 years and ≥ 10 years) and moderate wasting (yes/no) to remain in the model did not change the results. Boys were more likely to present with lipoatrophy (aOR: 4.3, 95% CI: 1.6–11.7) than girls. Children exposed to stavudine for ≥1 year had a greater risk for lipoatrophy than those never exposed (aOR: 3.8, 95% CI: 1.0–14.0), although the association was weak. Results were similar from a model including all cases of lipodystrophy (*n* = 33).

**Table 4:** Risk factors for lipoatrophy in HIV-infected children in the Maggsen cohort study.^a–b^ Dakar, Senegal

Characteristics	Univariable analysis^c^			Multivariable analysis^d^		
	OR	95% CI	*P* value	aOR	95% CI	*P* value
Boys vs. girls	4.3	1.6–11.6	0.004	4.3	1.6–11.7	0.005
Stavudine exposure						
≥ 1 year vs. no	3.6	1.0–12.6	0.04	3.8	1.0–14.0	0.04
< 1 year vs. no	1.5	0.5–4.8	0.48	1.4	0.4–4.4	0.59
Lopinavir/r exposure						
≥ 3 years vs. no	2.2	0.9–5.4	0.09	–	–	–
< 3 years vs. no	0.3	0.0–2.5	0.28	–	–	–
Moderate wasting^e^: yes vs.no	1.7	0.7–3.9	0.20	–	–	–
Age ≥ 10 vs. < 10 years	1.3	0.6–2.9	0.54	–	–	–

^a^Abbreviations: *aOR* adjusted odds ratio, *CI* confidence interval, *Lopinavir/r* lopinavir/ritonavir, *OR* odds ratio

^b^No missing for multivariable model

^c^Explanatory variables are included at *P* < 0.25 in multivariate analysis

^d^Explanatory variables are exited at *P* ≥ 0.20

^e^Moderate wasting (moderate acute malnutrition) is defined for both weight-for-height z scores (WHZ) in children < 5 years or body mass index-for-age z score (BMIZ) in children ≥5 years as being ≥ − 3 and < − 2 [20, 21]

## Discussion

The prevalence of clinical lipodystrophy was very low, 13%, in a cohort of HIV-infected Senegalese children and adolescents on ART for a median duration of 54 months. First, we observed only mild signs of lipodystrophy, by definition corresponding strictly to possible minor changes. Second, prior treatment with stavudine was limited in this Senegalese cohort, regarding both the proportion of children concerned, 18%, and the duration on treatment, eight months on average. However, children previously treated with stavudine for more than one year were at greater risk to present with possible sign(s) of lipoatrophy than those never exposed. Most studies are based on clinical examination for fat redistribution, which might be subjective. Some have coupled clinical assessment with various objective techniques including skin-fold thickness, dual energy X-ray absorptiometry (DEXA) and magnetic resonance imaging. These methods are prohibitively expensive and/or complex, if not unavailable, for routine use in low-income settings. Clinical assessment of fat redistribution in HIV-infected children is further complicated by the normal, dynamic alterations in body shape and composition occurring during childhood and adolescence. Hence some studies have considered only grades moderate to severe, because of uncertainty in providing a definitive diagnosis of mild grade lipodystrophy. Acknowledging the inherent uncertainty in the diagnosis of the mild grade, we found no instance of doubt in clinical diagnosis between mild versus moderate or severe signs of lipodystrophy.

There is overwhelming evidence that lipoatrophy is an adverse drug reaction to stavudine, with potential mechanisms including mitochondrial damage in adipocytes and inhibition of adipogenesis [24, 25]. Recent southern African studies reporting on the association between lipodystrophy and stavudine treatment in children are conflicting. Innes et al. found an alarmingly high prevalence, 36%, of moderate to severe lipoatrophy in prepubescent South African children after a median treatment duration of 41 months on stavudine [16]. They also reported that current treatment with stavudine was the predominant risk factor for lipoatrophy, as well as finding a cumulative effect, by year of exposure, on arm fat loss. Studies in Uganda and Tanzania reported lipodystrophy in 27% (mostly lipoatrophy, of all grades) and 30% (mostly mild lipoatrophy) of children aged two to 18 years [13, 15]. In the Ugandan study, involving children with a median of 3.8 years on ART of whom 20% had an ongoing exposition to stavudine, risk factors identified were older age, puberty onset and any exposure to stavudine [13]. Similarly, the Tanzanian study reported older age and longer exposure to stavudine as predictors of lipodystrophies in children under current exposure to stavudine for approximatively 3 years [15]. In 2012, most African children were still receiving stavudine-based ART. Thus a distinguishing feature between these studies and ours is the context of long-term and ongoing exposure to stavudine.

The effect of stavudine in causing lipoatrophy appears to be strongly dose-related [26]. The CHAPAS-3 trial, where stavudine was notably prescribed at lower doses [27] than in the previously cited studies, reported only 1% of lipodystrophy (lipoatrophies grade 2 and 3) in children previously treated with stavudine for a median of 3 years and randomized to continue with a stavudine-based regimen over 96 weeks [28].

In recent years, WHO reiterated recommendations for countries to phase out stavudine and today it is no longer considered in first-line options. Nonetheless, estimates suggest stavudine still contributed to between 7 to 9% of the NRTI procurement forecasted for pediatric use in low and middle-income countries in 2016 [29]. The early promotion of stavudine-sparing first-line regimen in Senegal since 2007 certainly contributed to limiting the occurrence of lipoatrophy in this cohort.

It had been suggested that zidovudine, which like stavudine, is a thimidine-analogue NRTI, could also lead to lipodystrophy, although to a lesser extent [13, 15, 30]. Zidovudine treatment was predominant in these Senegalese children’s ART regimen, and our results are consistent with those of the ARROW trial which reported, three years after randomization to lamivudine+abacavir+zidovudine, an extremely low rate of mainly mild lipoatrophy in children aged three to 19 years of less than 2% [31]. In that study, the use of stavudine (as a first-line substitution) and protease inhibitor (as second line) was minimal. In addition, substitution of stavudine with zidovudine significantly decreased severity or promoted resolution of mild to moderate lipoatrophy in HIV-infected Thaï children after 96 weeks [32] or more [33]. In the present study, thirty months had passed since the date of last individual exposure to stavudine and inclusion in the assessment. We suspect that a process of reversal of lipodystrophy figures occurred in this cohort. Together, these results provide reassurance on the substantially lower potential of zidovudine for lipoatrophy, in settings where stavudine has been massively replaced by zidovudine in first-line treatment.

The first generation of PI has also been associated with fat redistribution, both loss [12, 34, 35] and accumulation [11, 36, 37] in adults, and with similarly conflicting results in children [5, 7, 9, 38–40]. However, the role played by ART, and especially by the PI, in the pathogenesis of lipohypertrophy is less clear. The most accepted hypothesis is that PI impair adipocyte differentiation resulting in hypertrophy of adipose tissue, particularly in visceral tissue [2, 9, 12]. Although past and current exposure to lopinavir/r was substantial in this cohort, we did not find an association with lipodystrophy in multivariable analysis.

Ultimately, male sex was the main risk factor for lipoatrophy in the cohort. Boys were more likely to present with moderate wasting and a prepubescent status than girls at the time of the evaluation. Having excluded children with severe wasting from our study population, moderate wasting remained at rather high level, particularly among adolescents, and is consistent with figures reported from a recent study conducted in similar context in Mali [41]. Explanations for persistent wasting in the Senegalese adolescents on ART may lie in a therapeutic history of drug resistance development and late switch to second-line ART [19], a potentially high level of household food insecurity [42] and a substantial level of undernutrition in the general school population of the Dakar suburbs [43]. However, wasting was not associated with lipoatrophy in either univariable or multivariable analyses.

Some studies in high to middle-income countries reported that females were more likely to develop lipodystrophy than males [3, 7, 44]. In those settings, lipohypertrophy and combined type were more common than lipoatrophy. In recent studies reporting a high prevalence of lipoatrophy, sex was not identified as an independent risk factor [3, 15, 16]. Boys accumulate less subcutaneous fat mass than girls during childhood [45] and adolescence [46]. In addition, boys might have looked generally thinner than girls in this cohort. Combined, such considerations could have influenced the clinical assessment towards an overestimation of lipoatrophy in boys and/or under diagnosis in girls.

Lipodystrophies are frequently associated with metabolic disturbances, presented as a lipodystrophic syndrome [47]. It is recognized that PI may induce dyslipidemia, which may increase the risk of cardiovascular disease in adulthood [7, 38]. The rate of hypercholesterolemia was high, mainly led by elevated LDL-cholesterol, while hypertriglyceridemia was marginal in the cohort. As have others [13, 48], we found no association between lipodystrophy and dyslipidemia. Longitudinal analyses of blood lipid profile evolution are currently ongoing in the cohort (data not shown).

This study has several limitations. First, our cross-sectional study design does not allow for controlling the trend in evolution of reported lipodystrophy cases. However, the temporality of data, as well as the children’s profiles, suggest that the risk for an increase in prevalence or a development towards more severe forms of lipoatrophy are unlikely. Second, due to the small number of fat redistribution cases, a limited number of explanatory variables could be included in the multivariable model and statistical power might be limited. Third, the number of patients not included in this ANRS study cohort due to death or being lost to follow-up before enrollment is unknown, and the results might primarily apply to patients who remained in care.

## Conclusion

We report low prevalence of mild lipodystrophy in children and adolescents on long-term ART with limited exposure to stavudine. The responsiveness of the national Senegalese HIV programme to the 2007 WHO recommendations to phase out stavudine due to concerns over treatment toxicity, presumably helped to prevent the occurrence and/or the progression of lipodystrophy in these HIV-infected children and adolescents. Moreover, we found that consistent exposure to lopinavir/r and zidovudine did not lead to such adverse drug reaction in our cohort. These findings bring a reassuring message to clinicians in low-income settings where zidovudine is still massively prescribed in the NRTI backbone combination of pediatric ART and lopinavir/r is the most widely available PI.

## Abbreviations

aOR: adjusted odds ratio
ART: Antiretroviral treatment
BMIZ: Body mass index for age z-score
CI: Confidence interval
II: Integrase inhibitor
IQR: Interquartile range
Lopinavir/r: Lopinavir/ritonavir
NNRTI: Non nucleoside reverse transcriptase inhibitor
NRTI: Nucleoside/nucleotide reverse transcriptase inhibitor
OR: Odds ratio
PI: Protease inhibitor
RTV: Ritonavir
WHO: World health organization
WHZ: Weight for height z-score
